# Dementia in the Western Pacific region: From prevention to care

**DOI:** 10.1016/j.lanwpc.2022.100607

**Published:** 2022-09-26

**Authors:** The Lancet Regional Health – Western Pacific

Dementia is the collective term for neurological syndromes that are characterised by a clinically significant decline in an individual's cognitive, psychological, behavioural, or physical functioning. As a result, the affected individual gradually loses the ability to perform daily activities and there is no cure to reverse progression. The most common cause of dementia is Alzheimer's disease. Globally, the number of people with dementia has been predicted to increase from 57·4 million to 152·8 million in 2050. The increasing population with dementia and the associated cost and care burden have generated substantial interest in prevention, risk reduction, and delivery and quality of care for people with dementia.

The 2020 Lancet Commission on Dementia Prevention, Intervention, and Care showed that 40% of dementia cases are preventable if exposure to the following 12 known risk factors is eliminated: low education, high blood pressure, hearing impairment, smoking, midlife obesity, depression, physical inactivity, diabetes, social isolation, harmful use of alcohol, head injury, and air pollution. Although dementia cannot be eradicated by addressing these known risk factors alone, risk reduction strategies to improve education, nutrition, health care, and lifestyle have great potential to slow or even prevent the onset of the disease, which will ultimately lower the burden of dementia. In the Western Pacific region, the prevalence of dementia is expected to increase in many countries because of population ageing, especially in low-income and middle-income countries (LMICs). However, according to a study in The Lancet Public Health, the prevalence of dementia in high-income Asia Pacific countries is projected to reduce. This promising forecast is mainly attributed to the improvement of educational attainment and a decline in cardiovascular risk in those countries.

Tailored interventions for preventing dementia must be applied with an appropriate understanding of the major determinants in a specific population. By calculating the population-attributable fraction of the 12 potentially preventable risk factors for dementia, Etuini Ma'u and colleagues show that 47·7% of dementia cases are preventable in New Zealand; the rate is 40·8%, 47·6%, 51·4%, and 50·8%, for Asian, European, Māori, and Pacific peoples, respectively. Notably, major dementia risk factors in different ethnic groups are not the same. For instance, hearing loss, social isolation, and obesity are the major risk factors for European people; for Asian people, the top three risk factors are hearing loss, physical inactivity, and hypertension, whereas obesity, hearing loss, and low education have the highest population-attributable fraction for Māori and Pacific peoples. Earlier evidence suggests that the association between key risk factors and dementia is not significantly altered by race. The different prevalence and risk factors of dementia across different ethnic groups in the same country might be caused by underlying structural inequalities. For instance, rather than genetic differences, the high prevalence of risk factors such as obesity in Māori and Pacific peoples might reflect socioeconomic disadvantage and an increased vulnerability to obesogenic environmental influences. Furthermore, most modifiable risk factor studies are done in high-income countries. There might be other unmeasured risks factors for people living in LMICs or ethnic minority populations, which require further research to explore and validate.

During World Alzheimer's Month in September, 2022, Alzheimer's Disease International calls for more post-diagnostic support for people living with dementia and their families. People with dementia are highly dependent on carers and require complex health and social support from multiple sectors, such as primary health care, rehabilitation, residential care, and palliative care. One of the specific challenges in the Western Pacific region is inadequate preparedness of both human and financial resources for dementia care. For cultural and economic reasons, most people with dementia are living with and cared for by family members in the region, who often do not receive adequate information, training, or psychological and financial support from the community or the government. Liat Ayalon and Senjooti Roy discussed the role filial piety plays in ageism and elder care in Western Pacific countries. As a moral obligation, elder care faces challenges in practice due to modernisation and demographic shifts. Sole reliance on family members to provide care is not sustainable and cannot meet the needs of people with dementia. Furthermore, the stigma of dementia is common in many Asian countries, which prevents people from seeking diagnosis and care and puts more pressure on family members who are already overwhelmed by their daily duty. Countries in the region must develop and implement integrated and community-based care models that can be applied to different cultural settings and, at the same time, aid formal and informal carers with social and financial benefits to support their roles and reduce the stigma associated with dementia.

Despite the already large and increasing numbers of people with dementia living in the Western Pacific region, only four countries—Australia, Japan, New Zealand, and South Korea—have national dementia plans. To realise the vision of the WHO Global Action Plan on the Public Health Response to Dementia 2017-2025 to “have a world in which dementia is prevented, and people with dementia and their carers live well and receive the care and support they need to fulfil their potential with dignity, respect, autonomy and equality,” each Western Pacific country needs a national dementia plan that lays out how resources will be allocated to eliminate dementia risk factors, train and support carers, research new diagnostic and therapeutic modalities, and develop an inclusive society for people living with dementia and their families.

